# Ambulatory Management of Bite Injuries to the Hand: A Safe and Cost-Effective Option

**DOI:** 10.7759/cureus.62399

**Published:** 2024-06-14

**Authors:** Ibrahim I Haq, Bhagat Manku, Andrew Mahon, Clare Langley, Deepak Samson

**Affiliations:** 1 Trauma and Orthopaedics, University Hospitals Coventry and Warwickshire, Coventry, GBR

**Keywords:** cost-effective therapy, hand trauma management, hand trauma center, mammalian bite, dog bites wound

## Abstract

Introduction

Animal or human hand bites are a common presentation to the emergency department. If hand bites are not treated adequately, they can give rise to significant local and systemic complications, potentially leading to functional deficits that impact patients' lives.

Traditionally, hand bites require hospital admission for the administration of intravenous antibiotics and, in some cases, surgical intervention. A combination of the increasing incidence, hospital admission rates, and in-patient bed pressures prompted a change in our bite management protocol and a move toward ambulatory management of bite injuries. We found this new protocol to be safe, efficient, and cost-effective with a scope for wider implementation.

Aim

The primary outcome is to assess the feasibility of safely managing hand bites on an outpatient basis, by reviewing the local data before and after the change in practice. The secondary outcome is to compare the financial implications of treating hand bites with an outpatient approach.

Material and methods

All first-presentation adult consultations referred to Trauma and Orthopaedics from the emergency department over a three-month snapshot period were reviewed in 2017. This was repeated after the implementation of the updated handbite guidelines in 2023. Initial admission documentation as well as operation notes and clinic follow-up letters were each reviewed retrospectively.

Results

In 2017, 36 patients were identified over three months. The average time to surgery was 1.19 days with an average inpatient stay of 2.36 days. There were two re-operations and follow-up of two cases of osteomyelitis.

In 2023, 63 patients were identified over three months. The average time to surgery was 1.03 days with an average inpatient stay of 0.56 days. Thirty-seven surgeries were performed for 33 patients with 32% (20/63) of patients admitted directly from the emergency department. There were no documented cases of osteomyelitis on follow-up. The cost per patient episode decreased by 40% from 2017 to 2023, without accounting for inflation.

Conclusions

With the implementation of the new departmental guidelines, there has been a reduced average inpatient stay and reduced time to surgery without an increase in documented osteomyelitis. There is also a significant decrease in the average patient cost. This data suggests that without compromising patient safety it is possible to cost-effectively manage hand bites without the need for long inpatient stays.

However, it is imperative that there is close patient follow-up as well as prompt time to surgery to ensure patient safety. Our findings suggest a need for further research to strengthen the evidence supporting our conclusions.

## Introduction

Bites are increasingly common injuries, with the hand involved in a large proportion of them. There are 11 million pet cats in the UK, with 26% of households owning at least one cat [1]. With a dog population of 8.5 million in the UK (one for every seven to eight people), dog bites are the most common, although human bites (the so-called “fight bite") are also a major contributor to bite morbidity [2]. In our unit, we have also seen injuries caused by Gerbils, Rabbits, and also a Pike. The presentation of an animal bite injury is frequently delayed as many patients underestimate the injury owing to the innocuous initial appearance. The impact and burden, both logistic and financial, of bite injuries on the NHS is substantial.

Bite injuries to the hand have the potential to lead to a serious infection in addition to the structural damage caused, with the potential for longer-term morbidity of pain and stiffness. There are diverse pathogens involved, and small seemingly trivial wounds can progress to cellulitis, abscesses, septic arthritis, and osteomyelitis [3]. These conditions need inpatient admissions and sometimes prolonged antibiotic therapy. The importance of early recognition and initiation of treatment cannot be over-emphasized.

In a report published by the NHS England Digital, in the one-year period of 2021-2022, dog bites accounted for 8,819 finished inpatient admission episodes, which was a 33% increase from 2011 to 2012 [4]. A large proportion of these injuries need inpatient care and admissions just for dog bites, rising from 6.34 (95%CI 6.12-6.56) in 1998 to 14.99 (95%CI 14.67-15.31) admissions per 100,000 population in 2018 [5]. The strain on the system, both in terms of inpatient bed availability, and financial costs are considerable [6].

During a very busy winter season when the bed pressures in our large NHS hospital were enormous, we were challenged to consider the potential of a safe outpatient service. We adhered to the Virginia Mason Institute of Lean Philosophy and applied this thinking to our considerations [7]. In the context of increasing inpatient bed pressures and rising incidence and hospital admission of mammalian bite injuries, we present a pathway for ambulatory management of bite injuries. We believe this protocol has the potential to reduce the inpatient load and subsequent stress on the health care system, while not compromising on safety and efficacy. We consider that it would be straightforward to adopt widely through our NHS services.

## Materials and methods

In 2017, our standard management for animal bites to the hand called for inpatient admission and intravenous antibiotics for all animal bites followed by surgical debridement in the operating theatre as soon as possible. During this time, we only had access to hand trauma lists three times a week, which meant patients would often wait more than 24 hours for the first operation.

**Figure 1:**
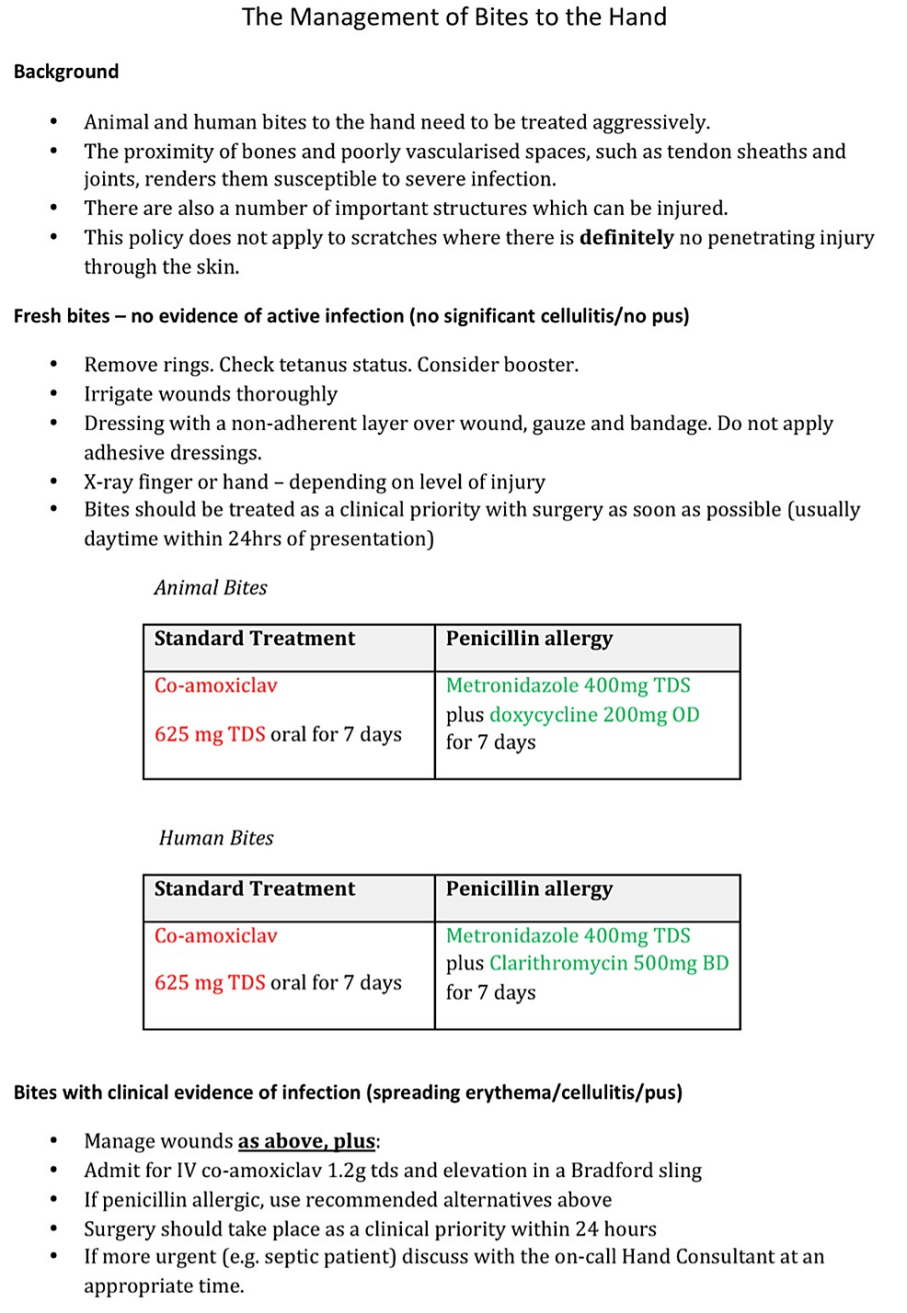
Local Updated Hand Bite Guideline The image is attributed to University Hospitals Coventry and Warwickshire.

A combination of the increasing incidence, hospital admission rates, and in-patient bed pressures prompted a change in our bite management protocol and a move toward ambulatory management of bite injuries. Following a discussion with our microbiology colleagues, we changed our management to a new protocol (Figure 1) consisting of thorough wound irrigation under local anesthetic in the emergency department and oral antibiotics prophylaxis for bites without overt clinical evidence of infection. As part of the new guidelines, our department was granted five theatre lists a week to allow for quicker time for surgery. Due to this change, patients were sent home and returned on the morning hand trauma list to be operated on as a day case procedure, being discharged on the same day except in the instances of a planned second look operation at 48 hours.

However, the guidelines did have a caveat that if there were bites with clinical evidence of infection such as spreading erythema, cellulitis, or frank pus they were to be admitted and treated with inpatient admission and intravenous antibiotics. Clinical assessment was done by doctors in the trauma and orthopedics team in the accident and emergency department to ensure the patient was safely assessed. 

Additional information was included in the guidelines to assist doctors in their assessments. This encompassed details such as obtaining appropriate radiographs and proper wound dressing techniques (Figure 1). The aim was to ensure that optimal treatment protocols were uniformly followed by doctors in the accident and emergency department.

We set out to evaluate the efficacy of our new animal bite management protocol with regard to patient safety, inpatient stay, time to surgery, complications, and cost efficacy. Additionally, we performed a cost analysis, evaluating the average expenses incurred per patient episode under each protocol with the costs not being adjusted for inflation. By comparing these costs across the two groups, we aimed to determine the financial impact and cost-effectiveness of the new management approach.

Our findings are intended to provide a better understanding of the benefits and potential drawbacks of the new protocol, ultimately guiding future improvements in animal bite management within our unit.

## Results

A total of 36 patients were treated under the original protocol between September and December 2017. Of these, two patients had unplanned re-operations, and two developed osteomyelitis. The average number of inpatient days was 2.36 and the average time to surgery was 1.19 days (Table 1).

**Table 1:** Primary and Secondary Outcome Data

	Sept to Dec 2017	Aug to Nov 2023
Number of patients	36	63
Bed days	2.36	0.56
Time to surgery (days)	1.19	1.03
Re-operations	2	0
Osteomyelitis	2	0
Average cost per patient episode	£2578	£1545

In 2023, 63 patients were identified over three months. The average time to surgery was 1.03 days with an average inpatient stay of 0.56 days. A total of 37 surgeries were performed for 33 patients with 32% (20/63) of patients admitted directly from the emergency department. There were no documented cases of osteomyelitis on follow-up (Table 1).

Our results showed that the number of bed days had reduced from just over two to approximately half a day. All the patients who required debridement were taken to theatre the following day on a hand trauma list so the time to surgery was dramatically shorter. From the patient's point of view, this meant more rapid care, a streamlined flow through the hospital, and a lower impact on their work and family lives. The cost per patient episode was 40% less than under the old protocol. 

## Discussion

The hand is the most common site of bite injuries [8]. Bite injuries to the hand have long been recognized as a major cause of morbidity and a subsequent strain on the health care system of a country.

The proximity of bones and poorly vascularized structures, like tendon sheaths and joints and numerous potential spaces, can lead to the oral flora of the biting animal causing significant infections even with small inoculating injuries [9]. Septic arthritis and osteomyelitis have been reported as a resulting sequelae [10]. Human and animal bites usually lead to polymicrobial infections with a mixture of aerobic and anaerobic organisms [11]. Pasturella and Eikenella species have been reported as the common organisms found in cat and human bites, respectively, while Staphylococcus, Streptococcus, and anaerobic bacteria are common in most bite wounds [8,12]. This knowledge is the basis of empirical antibiotic prophylaxis [13].

Patient factors can also influence the course and outcome of the injury with immunocompromised patients being especially susceptible to more severe infection. In addition, human bites can also transmit blood-borne viral (BBV) infections like HIV and Hepatitis B or C. Since 2019 our bite protocol has been further modified to assess for BBV infection risk as per NICE guidelines [14].

At the outset, the salient points of management are early recognition, evaluation, irrigation, antibiotic prophylaxis, and, if required, surgical debridement, and either primary or delayed repair of injured structures [15,16]. Existing protocols broadly follow these steps.

The results from our study show that with the new protocol, there were no unplanned re-operations or complications such as osteomyelitis. There was a significant reduction in the number of bed days and the time to surgery. As a consequence of the more streamlined process, the patient flow through the system was more efficient. This manifested as a cost saving of more than 40% without adjusting for inflation per patient episode (approximately £400,000.00 per annum) and more importantly, improved the patient experience as well. From a financial standpoint, there is a huge incentive for NHS trusts and hospitals to take this approach as it frees up hospital beds and keeps costs down with no evidence of reduced patient safety.

In the same time period in 2019, under the new treatment protocol, 37 patients were treated with bite injuries to the hand. Of these, three presented with overt infection and required inpatient admission. One patient had a planned re-operation at 48 hours for a second look; however, there were no unplanned re-operations and no late complications.

## Conclusions

With the implementation of the new departmental guidelines, there has been a reduced average inpatient stay, reduced time to surgery without an increase in documented osteomyelitis, and a significant decrease in the average patient cost.

In the setting of access to regular trauma lists, our protocol for ambulatory management of bite injuries provides an efficient, safe, and cost-effective option that we believe would be straightforward to implement across the NHS. Our findings suggest a need for further research to strengthen the evidence supporting our conclusions.
